# PGJ_2_ Provides Prolonged CNS Stroke Protection by Reducing White Matter Edema

**DOI:** 10.1371/journal.pone.0050021

**Published:** 2012-12-20

**Authors:** James D. Nicholson, Adam C. Puche, Yan Guo, Daniel Weinreich, Bernard J. Slater, Steven L. Bernstein

**Affiliations:** 1 Department of Ophthalmology and Visual Sciences, University of Maryland-Baltimore School of Medicine, Baltimore, Maryland, United States of America; 2 Department of Anatomy and Neurobiology, University of Maryland-Baltimore School of Medicine, Baltimore, Maryland, United States of America; 3 Department of Pharmacology, University of Maryland-Baltimore School of Medicine, Baltimore, Maryland, United States of America; Massachusetts General Hospital/Harvard Medical School, United States of America

## Abstract

Few clinically effective approaches reduce CNS-white matter injury. After early in-vivo white matter infarct, NFκB-driven pro-inflammatory signals can amplify a relatively small amount of vascular damage, resulting in progressive endothelial dysfunction to create a severe ischemic lesion. This process can be minimized by 15-deoxy-Δ^12,14^-prostaglandin J2 (PGJ_2_), an analog of the metabolically active PGD_2_ metabolite. We evaluated PGJ_2_'s effects and mechanisms using rodent anterior ischemic optic neuropathy (rAION); an *in vivo* white matter ischemia model. PGJ_2_ administration systemically administered either acutely or 5 hours post-insult results in significant neuroprotection, with stereologic evaluation showing improved neuronal survival 30 days post-infarct. Quantitative capillary vascular analysis reveals that PGJ_2_ improves perfusion at 1 day post-infarct by reducing tissue edema. Our results suggest that PGJ_2_ acts by reducing NFκB signaling through preventing p65 nuclear localization and inhibiting inflammatory gene expression. Importantly, PGJ_2_ showed no *in vivo* toxicity structurally as measured by optic nerve (ON) myelin thickness, functionally by ON-compound action potentials, on a cellular basis by oligodendrocyte precursor survival or changes in ON-myelin gene expression. PGJ_2_ may be a clinically useful neuroprotective agent for ON and other CNS infarcts involving white matter, with mechanisms of action enabling effective treatment beyond the currently considered maximal time for intervention.

## Introduction

Prolonged CNS ischemia causes microvasculature injury, producing tissue edema, micro-thrombus and hemorrhagic conversion [1]. Post-infarct edema compromises capillary function [1]–[4]. Edema reduction, independent of other therapy types, is therefore likely to be useful in CNS ischemia treatment.

Much attention focuses on grey matter (GM) ischemia, but relatively little on isolated white matter (WM) infarct. WM comprises 40–44% % of the human brain [5], but 13–15% of the rodent brain [6]. The smaller WM volume makes WM ischemia challenging to study using *in vivo* rodent models. While WM total oxygen requirement is less than that for GM [7], the quantitative vascular differences between the two suggest that WM may be equally sensitive to microvascular injury that results in long-term capillary dysfunction.

The mammalian optic nerve (ON) is a myelinated WM tract possessing all the essential attributes of other CNS WM regions. Unlike other CNS tracts, the ON has an isolated vascular supply [8]. Optic nerve axons derive from the retinal ganglion cell (RGC) neurons whose cell bodies are in the retina of the eye. Nonarteritic anterior ischemic optic neuropathy (NAION) is a sudden idiopathic ON ischemic event in the anterior ON that results in edema and permanent ON damage [9]. A rodent NAION model has been generated [10] (rodent anterior ischemic optic neuropathy; rAION), using laser light activation of intravenous rose Bengal (RB) dye within the target capillary field. The rAION model injures the smallest ON capillaries, sparing larger vessels, and resulting in ON edema [10]. Previous studies that have revealed early edema-associated capillary changes following CNS ischemic lesions were performed in cortical grey matter or analyzed as part of a complex lesion such as a middle cerebral artery occlusion (MCAO) [11], [12]. We utilized the ON to examine microvascular changes following WM ischemia using a newly devised quantitative vessel filling technique.

Prostaglandin J_2_ (PGJ_2_) is a compound derived from the nonenzymatic modification of prostaglandin D_2_ (PGD_2_) and has been found to be neuroprotective in a number of systems [13], [14]. PGJ_2_ binds to and activates peroxisome proliferator-activated receptor-gamma (PPAR-γ) and down-regulates nuclear factor kappa B (NFκB) [15]. Both these pathways down-regulate inflammation [16]. Post-infarct PGJ_2_ administration is neuroprotective following middle cerebral artery occlusion (MCAO) by non-overlapping NFκB and PPAR-γ dependent and independent mechanisms [14], [17] as well as being anti-inflammatory when used pre-induction in models of mixed stroke and in non-neuronal systems.

PGJ_2_ reportedly stabilizes vascular capillary flow (for review see [18]), but is also reported to be toxic when tested *in vitro* against neuronal cells [19], as well as oligodendrocytes and their precursors [20]. However, *in vitro* analysis is subject to a number of caveats, most notably the enhanced PGJ_2_ elimination found *in vivo* and the extended exposure time to high concentrations of PGJ_2_ found *in vitro*. Thus the question of whether such a potentially useful compound is too toxic to use for ischemia treatment should be answered in an intact system using reasonable doses and route of administration suitable for a neuroprotectant. We found that PGJ_2_ is effective in protecting against white matter ischemia-induced damage without apparent effect on oligodendrocyte function or oligodendrocyte precursor development post-treatment. Our data suggests that PGJ_2_ may be a clinically effective, non-toxic *in vivo* therapeutic adjunct in reducing damage following isolated white matter stroke by directly reducing edema.

## Methods

### Animals

This study was carried out in strict accordance with the recommendations in the Guide for the Care and Use of Laboratory Animals of the National Institutes of Health. The protocol was approved by the Animal Care and Use Organization (ACUO) of the University of Maryland at Baltimore (#0908001; Institutional approval A3200-001). All surgery was performed under Ketamine-Xylazine anesthesia, and all efforts were made to minimize suffering. Male Sprague-Dawley (SD) rats (150–200 g) were obtained from Harlan Laboratories, USA.

### Optic Nerve Stroke and treatment

ON infarct was induced as previously described [10]. Briefly, intravenous (i.v.) Rose Bengal (RB) (Sigma-Aldrich; 2.5 mg/kg in sterile saline) was administered via tail vein. RB was activated by intraocular optic nerve illumination via 532 nm wavelength laser light by illuminating the optic disk with a 500 µm spot size at 50 mW intensity for 12 seconds. This results in ON ischemia at 1 day with ∼55% loss of retinal ganglion cells by 30 days post-induction [21]. 15-deoxy-Δ^12,14^-Prostaglandin J_2_ (PGJ_2_) was purchased from Cayman Chemicals (Ann Arbor, MI). resuspended in 20% ethanol-0.9% saline and sterile filtered prior to intravenous injection. Animals received either vehicle (20% ethanol-0.9% saline) or a single dose of PGJ_2_ i.v. via tail vein post-induction.

### *In vivo* imaging

ON edema and the retina were evaluated in the living animal using a plano-convex contact lens, enabling visualization of the rodent retina and anterior optic nerve (Bernstein et al. 4153-62). Optic nerve fundus images were obtained at baseline and one day post-induction using a digital camera (Nikon D1X). We also visualized and evaluated the retinal cell layers and ON diameter via a spectral domain-optical coherence tomograph (SD-OCT) (Heidelberg Instruments), using the same contact lens. ON edema quantification was performed by measuring the width of the optic nerve shadow (defined as the region between the ends of the retinal image) at the greatest scanned point.

### RNA isolation and quantitative real-time polymerase chain reaction (qRT-PCR)

The proximal 3 mm of the ON was isolated and stored at −80°C. Total ribonucleic acid (RNA) was isolated using the RNeasy micro kit (#74004, Qiagen Inc.) and deoxyribonuclease-1 treated to eliminate genomic DNA contamination. Nerves were frozen on dry ice, then homogenized in lysis buffer using linear acrylamide as a carrier, followed by proteinase K digestion. RNA was analyzed for purity and quality using an Agilent Bio-analyzer. Because of the low yield for individual ON samples, we used the single chimeric primer amplification (SPIA) method (Ovation pico system, Nugen Corp.) to provide non-biased linear amplification of small amounts of mRNA. This reduced animal use, enabling us to compare gene response in individual rats for some studies rather than using pooled mRNA. For ON toxicity studies, RNA preparations were prepared from pooled (n = 6 optic nerves), and random primed complementary DNA (cDNA) was generated without an initial linear amplification step. RNA was converted to first strand random primed cDNA via RETROscript 1710 kit (Ambion Inc.). cDNA was used for qRT-PCR with gene-specific primers using Syber green dye (Bio-Rad Laboratories), with both cyclophilin B and β-Actin expression used as internal controls. Specific mRNA levels were evaluated using the ddCT method of difference subtraction from control gene expression levels. The following primers were used: Tumor necrosis factor *alpha* (TNFα) (f) actcccagaaaagcaagcaa, (r) cgagcaggaatgagaagagg; interleukin 1-*beta* (IL-1β) (f) gctagggagcccccttgtcg, (r) gctctgagagacctgacttggca; cyclophilin B (f) tgacggtcaggtcatcactatc, (r) ggcatagaggtctttacggatg. NG2 proteoglycan (f) acccaggctgaggtgaatgctg (r) tggcagccagatccctcgca (127 bp) Myelin basic protein (MBP) (f) gcgacgcagttgcctgggat (r) cgcgatgctcgcgggtacaa (138 bp) IL-4: (f) (gatgggtctcagcccccacctt (r) ccgtggataccgttcccggtac (72 bp) platelet derived growth factor *alpha* (PDGFRa) (f) agacacagctcgcagacttcgga (r) tccgagcctgccagttacagga (170 bp) Olig1 (f) gcgacgcagttgcctgggat (r) cgcgatgctcgcgggtacaa (138 bp) Olig2 (f) accagcggaaccccgaaagg (r) agaacctggctctgggcgct (73 bp).

### Compound action potentials (CAPs)

CAPs were recorded from rat ONs harvested immediately after CO_2_ euthanasia. ONs were dissected and immediately submerged in ice-cold (4°C) Locke's solution composed of (in mM): 136 NaCl, 5.6 KCl, 14.3 NaHCO_3_, 1.2 NaH_2_PO_4_, 2.2 CaCl_2_, 1.2 MgCl_2_, 11 mm dextrose, equilibrated continuously with 95% O_2_, 5% CO_2_, pH 7.2–7.4. Nerves were pinned to the Sylgard (Dow Corning)-coated floor of a recording chamber (∼0.25 ml volume) and superfused (3–5 ml/min) with oxygenated Locke solution at room temperature, 22–24°C. CAPs were recorded with a glass suction electrode connected to the input stage of an alternating current–coupled differential preamplifier (0.1–1 kHz model DAM-5A, World Precision Instruments). Data were filtered at 2 kHz and sampled at 10 kHz. CAPs were evoked with electrical pulses (0.1–0.5 msec in duration) elicited at 0.2 Hz using a second glass suction electrode. CAPs were digitized via a Digidata 1200 A/D converter (Axon Instruments) and stored on a computer. Ten CAPs were averaged before saving. Data acquisition and storage were controlled via pClamp 9.1 (Axon Instruments) and analyzed with Clampfit 8.2 software (Axon Instruments).

### ON vascular filling quantification

Quantitative ON vascular analysis was performed using tissue from terminally anesthetized (surgical plane anesthesia) rats. Animals were placed on a warming pad (∼38°C) and transcardially perfused sequentially with the following heated (∼38°C) solutions: 120 ml heparinized saline (50 units/ml) with 2 µg/ml atropine sulfate (Sigma Chemicals) and 100 µM adenosine (HAAS solution), 50 ml fluorescein-conjugated bovine serum albumen (FITC-BSA) with 2% dissolved gelatin (300 bloom, Sigma Chemicals) in HAAS solution, 20 ml FITC-BSA with 4% dissolved gelatin in HAAS solution. The descending aorta was clamped during perfusion. The ascending aorta was clamped under pressure at perfusion's end and the carcass immersed in ice water slush for 10 minutes to set the gelatin. A three-way valve with syringes was used to perform the perfusions with the inclusion of a 1.0 µm filter (#6821-1310, Whatman) added inline to remove un-dissolved gelatin. Tissues were fixed in 4% paraformaldehyde (PFA), washed in phosphate buffered saline (PBS), embedded in 10% gelatin (#G1890, Sigma), and re-fixed for an additional 24 h in phosphate buffered saline-4% paraformaldehyde (PFA). Tissues were cryosectioned to 40 µm and imaged by confocal microscopy using tiled z-stacks from a Zeiss LSM510 Duo fitted with a 40× 1.4 NA oil objective. Vascular data was quantified using a filament model constructed in Imaris software (Bitplane Software). FITC-BSA was created and purified in our lab by reacting fluoroscein isothiocyante (Sigma) with fraction V BSA (Sigma) and dialyzing against saline until no unreacted fluorescein was evident. The solution was then concentrated 2× using a size-exclusion spin column with a 30 kDa molecular weight (MW) cutoff and stored aliquoted at −80°C until use.

### Confirmation of ON edema at site of rAION lesion-blood brain barrier (BBB) breakdown

The vasculature of terminally anesthetized rats 1 d post-rAION induction were cleared with saline-heparin, and perfused with a mixture of rhodamine-bound 3000 kDa MW dextran (Sigma-Aldrich), and FITC-BSA (66,000 kDa) in 2% gelatin. The aorta was clamped, and animals were immersed in ice water. Tissues were isolated and post-fixed in 4% PF-PBS, sectioned at 30 microns, and examined by confocal microscopy using a Z-step function to merge the images, by neuroleucida version 6 software (Microbrightfield; Williston,VT).

### Retinal and ON immunohistochemistry

Retinal ganglion cell (RGC) stereology was performed on retinae from paraformaldehyde (PFA)-fixed eyes. Retinae were isolated and permeabilized by freezing, digested with hyaluronidase (Sigma-Aldrich) and incubated with goat polyclonal antibody to Brn3a (sc-31984, Santa Cruz Biotechnology). ON edema and BBB-serum leakage were assessed in ON sections using 1∶2000 cy3-conjugated donkey anti-rat IgG (Jackson Immunoresearch). We also utilized a mouse (GA5) antibody to glial acidic fibrillary protein (GFAP) (cell signaling, cat #3670).

### NFκB quench immunoassay

This was performed on 40 um thick ON floating sections. The p65-NFκB subunit was detected using a rabbit anti-p65 antibody (sc-372, Santa Cruz Biotechnology) at 1∶4000. Sections were incubated 1∶2000 in biotinylated donkey anti-rabbit IgG (Jackson Immunoresearch), washed in PBST and developed using nickel-diaminobenzidine (Ni-DAB), using the Elite ABC kit (Vector Labs). Sections were counterstained with DAPI (1∶20,000)

### Western analysis

ONs and corpus callosum (CC)-white matter tract were pooled from three animals and homogenized in radio-immunoprecipitation assay buffer (Amersham Biosciences). Five micrograms of denatured protein/lane was loaded on 4–15% polyacrylamide gels, electrophoresed, transferred to polyvinyllidene fluoride (PVDF) membrane, and detected using secondary antibodies coupled to horseradish peroxidase (Vector labs) and developed using Lumi-light substrate (Roche Laboratories). Antibodies used included the NFκB- p65 subunit (sc372, Santa Cruz Biotechnology, CA), an antibody that cross-reacts with p50/p65-NFκB subunits p65/p50 (#4764, Cell Signaling), and inducible nitric oxide synthase (iNOS) (#160862, Cayman Chemical). β-Actin (#A5441, Sigma) and Lamin C (#4777, Cell Signaling) were used as loading controls [22].

### Retinal ganglion cell neuron (RGC) Stereology

Stereology was performed on flat-mounted whole retinae 30 days post-induction, using StereoInvestigator software (Microbrightfield Inc.) on a Nikon E800 microscope with a motor-driven stage driven by the stereological software to generate random fields of up to 30 cell nuclei for counting. A minimum of 1000 cells Brn 3a(+) were counted per retina, which is greater than the number required by the Schmitz-Hof equation [23] for statistical validity.

### ON-oligodendrocyte precursor Stereology

Confocal images were acquired in tiled z-stacks on an LSM-510 duo microscope (Zen software, Zeiss Inc.). Using Imaris software (Bitplane Inc.), an isosurface was created within the z-stack on GFAP+ (Cy3 channel) regions of the ON to calculate tissue volume. The isosurface was used to mask the NG2-Cy5 channel, setting all voxels ouside the mask to zero. Both NG2(+)/GFAP(+) (considered to be astrocyte precursors) and NG2(+)/GFAP(−) (oligodendrocyte precursors) were detected. NG2(+)/GFAP(−) (cell body and processes) cells with a DAPI positive nucleus present within the masked volume were counted as positive oligodendrocyte precursors.

### Transmission electron microscopy

Sections of optic nerve prepared as above for vascular imaging were further analyzed after embedding in Durcupan resin (Electron Microscopy Sciences, PA). Specimens were fixed in 2% paraformaldehyde, 2.5% glutaraldehyde, 2 mM calcium chloride in 0.1 M Phosphate buffer (pH 7.4). and post-fixed with 1% osmium tetroxide, 1% potassium ferrocyanide in 0.1 M phosphate buffer. Specimens were washed and dehydrated using increasing ethanol washes, followed by 1% uranyl acetate and finally infiltration with Durcupan resin and polymerized at 60°C overnight. Ultrathin (70 nm) sections were cut and collected using a Leica UC6 ultramicrotome (Leica Microsystems, Inc., Bannockburn, IL), counterstained with uranyl acetate and lead citrate, and examined in a transmission electron microscope (Tecnai T12, FEI Inc.) operated at 80 kV. Digital images were acquired using an AMT bottom mount CCD camera and AMT600 software (Advanced Microscopy Techniques, MA).

Myelin quantification was performed on 3 optic nerves/treatment group, 30 days post-treatment. 5 axons from each size family (small, medium, large diameter axons) were identified at low magnification, and myelin thickness was measured at the narrowest point for each axon at high (30–40,000×) magnification. Myelin values were averaged for each group.

## Results

### PGJ_2_ reduces ON edema

**Figure 1:**
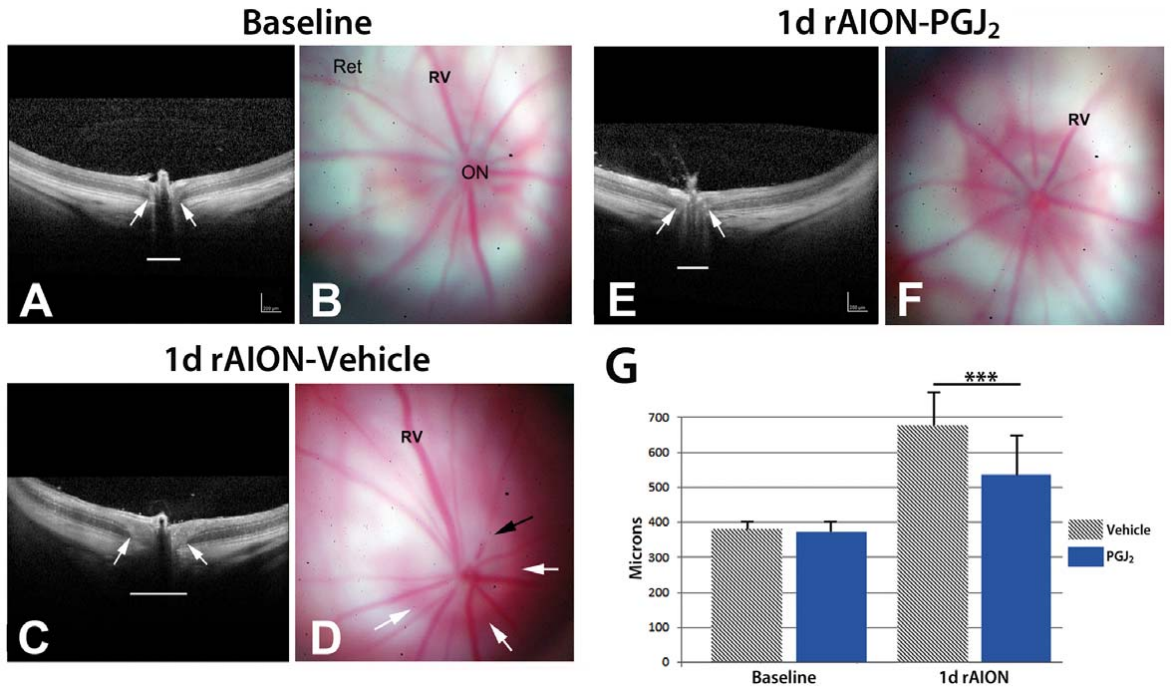
SD-OCT and retinal color photos of naïve, and rAION-induced vehicle and PGJ_2_ -treated eyes. A,B. Naïve eye. By OCT, the retina is flat against the back of the eye. The ON shadow is narrow (arrows and white line). Retinal photo reveals the ON is flat against the retina, with no protrusion. Retinal veins (Rv) are of narrow caliber. C,D. rAION-vehicle treated. ON shows increased diameter by OCT analysis (arrows), compared with the naïve eye (compare white line lengths). The retinal photo reveals a swollen and edematous ON (delineated by the white arrows). Crossing vessels are obscured in the edematous nerve (black arrow). E,F. rAION- PGJ_2_ treatment. The ON shadow by OCT imaging is smaller, compared with the vehicle treated animal (Compare arrows and underlying diameter line in OCT photos of naïve, and vehicle- and PGJ_2_-treated, rAION induced eyes). ON edema in PGJ_2_-treated animals is reduced in the color photo and is similar to the naïve eye. Retinal veins (Rv) engorgement is reduced. G. SD-OCT quantification. The optic nerve diameter is equivalent in both naïve and uninduced PGJ_2_-treated animals. One day post-induction, the ON diameter is increased in both groups. ON diameters of PGJ_2_-treated animals are narrower, compared with vehicle-treated animals. The PGJ_2_-associated edema reduction is statistically significant (*** p<0.05; (n = 7), Bonferroni multiple comparison test).

Naïve rats have an intraocular ON segment that is flat, with a reddish hued border (Figure 1B). Retinal veins (Figure 1B: Rv) are of normal caliber. One day after rAION-induced infarct, the ONs of vehicle treated animals (Figure 1D) are edematous and increased in size (Figure 1D), with whitening and blurred margins, compared with naïve animals (compare Figure 1D, white arrows, with Figure 1B). Retinal veins are engorged, with edema blocking vein outlines at the ON margin (Figure 1D, black arrow). The ON head one day post-infarct in animals treated acutely with PGJ_2_, is reduced in size, with reduced edema (compare Figure 1F, PGJ_2_ treated, with vehicle treated, Figure 1D.

We confirmed *in vivo* changes in post-infarct ON edema by spectral domain-optical coherence tomography (SD-OCT). SD-OCT utilizes infra-red laser imaging to identify subtle alterations in tissue density, thickness and disruptions. SD-OCT can reveal cross-sectional tissue features and resolve questions concerning sub-surface structural changes in living tissue. SD-OCT data are shown in Figure 1. The retina in naïve eyes is visualized as a laminated structure (Figure 1A, ret), The ON diameter is indicated (Figure 1A, white arrows). One day post-rAION in vehicle treated animals (Figure 1C) there is increased opacity of the intra-retinal axonal layer and ON diameter expansion (Figure 1C, arrows). In contrast, SD-OCT of rAION-induced PGJ_2_-treated animals show reduced ON edema and diameter (Figure 1E) compared with vehicle treated rats (arrows Figure 1E; compare with 1C). To confirm these findings, we quantified the changes in ON edema, using the SD-OCT. ON widening was compared in animals either treated with PGJ_2_ (n = 5 animals) or vehicle (n = 5 animals) (Figure 1G). There was reduced ON edema 1 d post-rAION induction in the PGJ_2_-treatment group (mean ON width 535.8±108 microns), compared with vehicle-treated animals (649.8±150.5 microns), while there were no differences in baseline ON widths of either group (372.8±13.3 PGJ_2_ treated v. 378.6±22.2 vehicle-treated) These results confirm that that PGJ_2_ treatment reduces ON edema following infarct.

### PGJ_2_ improves capillary perfusion by reducing post-infarct edema

We quantitatively evaluated capillary perfusion volume in the ON proximal segment (the region directly affected by the ON infarct). Capillary volumes were compared post-infarct in vehicle *vs*. PGJ_2_-treated eyes. We used the capillary volume of the un-infarcted eye of each animal as an internal control to eliminate individual variation in capillary density, which can be considerable.

**Figure 2:**
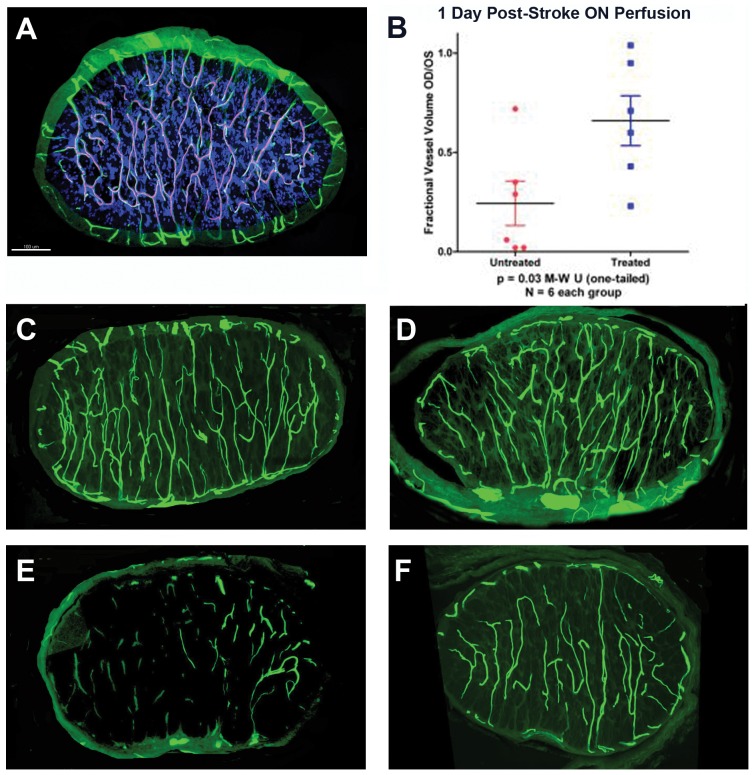
Microvascular analysis of naïve and infarcted ONs at 4 hours and 1 day post-induction. A. FITC-BSA vascular imaging of naïve ON. Filled capillaries are relatively uniformly distributed throughout the nerve, communicating with peripheral vasculature. B. Quantitative capillary analysis of 1 day post-rAION-induced ON, in vehicle- and PGJ_2_-treated animals. Infarcted nerves were compared with the contralateral (naïve control) nerve of the same animal (1.0 of filling), and expressed on the Y axis as a fractional vessel volume (OD/OS). PGJ_2_-treated animals show significantly more patent capillaries at one day than vehicle treated animals (Mann-Whitney U test, p<0.03). C and D: ON capillary filling 4 hours post-induction. There is minimal loss of capillary patency in both vehicle- (panel C) and PGJ_2_-treated (panel D) nerves. E and F: ON capillary filling 1 day post-induction. There is significant loss of vascular patency in ONs of vehicle treated animals (panel E). ONs in PGJ_2_-treated animals (panel F) reveal considerably more patent vasculature at one day. Scale bar: 100 microns.

Our analysis revealed that infarct-related primary ON damage is associated with decreased capillary perfusion, which occurs in the anterior 1 mm of the ON and is the site of the rAION primary lesion [24]. This region of the naïve ON has a dense anastomotic capillary network with limited arterial perfusion or venous drainage (Figure 2A) [8].

The naïve ON has an extensive capillary network that is relatively uniformly distributed throughout the tissue (Figure 2A). Four hours post-rAION there is a detectable but minimal capillary dropout in both the vehicle (Figure 2C) and PGJ_2_-treated (Figure 2D) ONs. No significant differences were found in contralateral (uninduced) ONs between PGJ_2-_ and vehicle-treated animals (data not shown), when compared with naïve contralateral eyes. One day post-rAION, vehicle-treated animals showed a severe loss of capillary vascular perfusion (Figure 2E), compared with PGJ_2_-treated animals (Figure 2F). Quantified mean perfusion of vehicle treated ON capillaries was only 25% of filling compared with contralateral un-infarcted control ONs (Figure 2B, untreated). In contrast, PGJ_2_-treated animals had dramatically more ON vascular perfusion at the level of the infarct, with a mean of 65% of un-infarcted nerve perfusion (Figure 2B, treated). This difference was significant (p<0.03 by single-tailed Mann-Whitney U test).

Capillary nonfilling can be due to intracapillary blockade by fibrin, intracellular edema and/or capillary compression caused by capillary leakage and extracellular edema.

**Figure 3:**
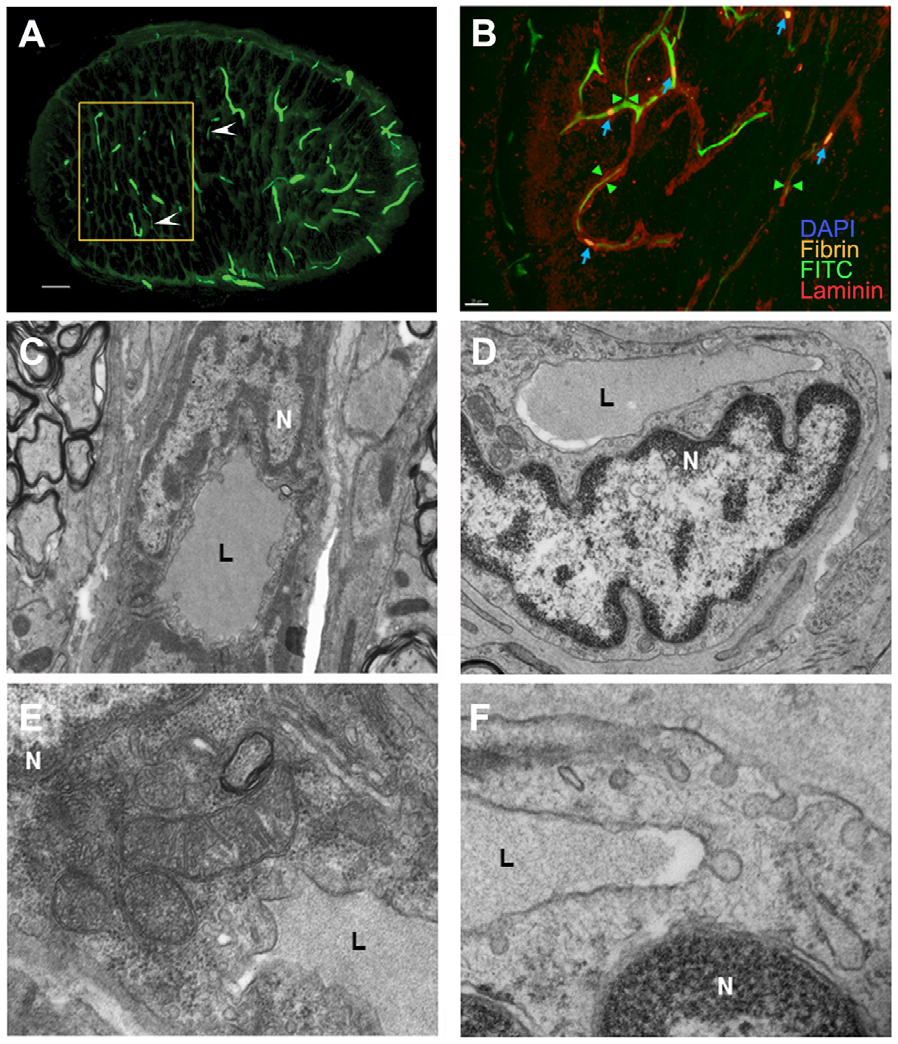
Immunohistochemical and ultrastructural analysis of vascular damage following ON infarct. A–D: Immunohistochemical imaging of vessel and luminal changes. A. FITC-BSA filling of ON 1 day post-infarct. There is general loss of capillary filling. Thinned areas of capillaries are indicated by arrowheads. The area analyzed ultrastructurally (by TEM, panels D and F) is indicated by the rectangle. There is incomplete filling of the capillary bed, compared with naïve controls. B. Immunohistochemical analysis of capillary changes 1 day post-infarct. Immunostaining with laminin (red) and fibrin (orange). FITC-BSA vascular filling (green) is incomplete, with occasional small fibrin-fibrinogen thrombi (indicated by blue arrows) present in the capillaries, which co-localize with reduced and absent areas of capillary patency. C–F: ultrastructural analysis using TEM. C. Naïve ON capillary, magnification 4400×. An endothelial cell nucleus (N) is adjacent to the capillary lumen (L). D. ON capillary 1 day post-induction, magnification 4400×. The endothelial cell nucleus (N) is lobulated and enlarged, and protruding into the lumen (L), which is reduced in diameter. E. Naïve ON capillary, magnification 15000×. Mitochondria are intact and there are small vacuoles in the endothelial cytoplasm, adjacent to the lumen (L). The nucleus (N) is normal in appearance. F. ON capillary 1 day post-induction, magnification 15000×. The endothelial cell nucleus (N) has changed appearance. Large cytoplasmic vacuoles, emptying on both sides of the cell membrane, suggest enhanced fluid transit from the lumen and increased extracellular fluid. Scale bars: A. 50 microns. B. 10 microns.C,D. E,F.

We analyzed capillary perfusion in the ON capillaries of both uninduced and rAION-induced animals one day post-induction, using a number of complementary approaches to identify the various components. We evaluated the presence of fibrin using an antibody to fibrin-fibrinogen. FITC-BSA capillary filling provided an indication of vascular patency and thrombosis. Total capillary networks were localized using an antibody for laminin, which recognizes capillary basement membranes. These results are shown in Figure 3.

ON capillary filling (by FITC-BSA), in a vehicle-treated animal 1 day post-induction, is shown (Figure 3A). Thinning (Figure 3A, arrowheads) and loss of FITC-filled capillary lumina are apparent. At higher magnification (Figure 3B) shows combined FITC-BSA filling with fibrin-laminin immunohistochemical analysis of a similar ON 1 day post-induction. Occasional focal fibrinogen signal (indicative of fibrin clots, blue arrows) is present within the capillaries (Figure 3B, arrowheads). Laminin immunofluorescence was apparent without co-localization of fibrinogen or FITC-BSA filling. Thus, this form of ischemic ON induction results in both edema-associated capillary collapse, as well as a minor component of fibrin thrombi.

Capillary collapse was further analyzed by ultrastructural analysis of a portion of a vehicle-treated ON one day post-induction (the area outlined in Figure 3A by a yellow box). We compared these results with the features of naïve ON capillaries. These results are seen in Figure 3C–F.

TEM-ultrastructural analysis of naïve ON (Figure 3C and 3E) were compared with rAION-induced ON 1 day post-induction (Figure 3D and 3F). In naïve ON, the capillary lumen (Figure 3C, ‘L’) is patent and filled with FITC-BSA. In comparison, the nuclei of capillary endothelial cells in an ON region affected by rAION are swollen (Figure 3D), with constriction of the capillary lumen (L), suggesting the presence of cytotoxic edema (compare Figure 3C with 3D). At high magnification, the naïve ON capillary shows intact mitochondria and the presence of small vacuoles (Figure 3E). In comparison, vacuoles fusing with the cytoplasmic membrane are consistently present in the cytoplasm of rAION-induced endothelial cells (Figure 3F), which communicates with both the lumen and extracellular basement membrane surfaces. Thus, the observed loss of perfusion involves the direct collapse of capillaries due to swelling endothelial cells, and possibly the production of extracellular edema due to extravasating blood serum.

**Figure 4:**
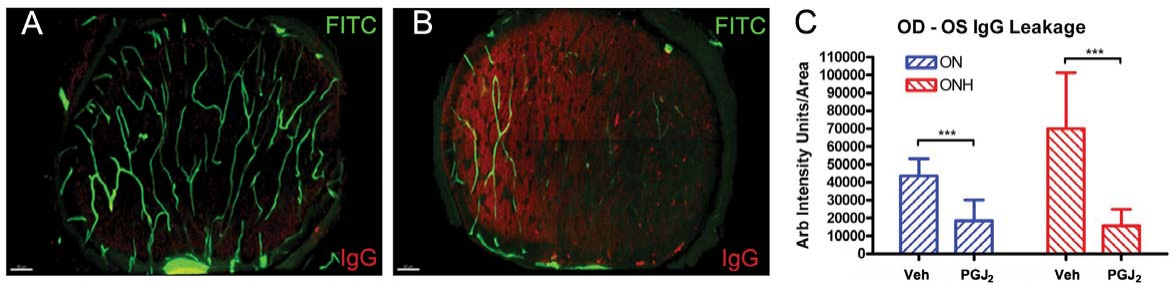
BBB breakdown measured by serum IgG leakage in FITC-BSA filled ON. Animals were perfused with FITC-BSA (green). ONs were fixed and reacted with anti-rat IgG (red), as a marker of serum leakage. Two ON regions were evaluated: the optic nerve head (ONH), which is the region of the primary injury and ON ∼2 mm posterior (ON). A. Naïve eye. There is minimal IgG signal in the ON cross-section. Vascular perfusion is intact, as evaluated by FITC-BSA signal. B. One day post-induction. There is reduced FITC-BSA signal, and increased IgG signal. C. Quantitative analysis of intraneural IgG levels 1 d post-rAION. There is increased IgG signal in the primary lesion site (the optic nerve head, ONH) and more posteriorly, in the ON, in animals treated with vehicle, compared with PGJ2-treated animals. This difference is significant (***; Mann-Whitney U-test, p<0.05). Scale bars: 50 microns.

We also evaluated blood brain barrier (BBB) breakdown and related interstitial tissue edema by staining and quantifying the presence of endogenous rat IgG in the ON substance of naïve and rAION-induced animals (Figure 4). The un-infarcted ON of a vehicle-treated animal (Figure 4A) has minimal endogenous rat IgG signal while the rAION-induced ON of the same animal at 1 d revealed diffuse IgG staining in the affected area (Figure 4B), implying the occurrence of BBB breakdown and serum protein extravasation. Quantification of the fluorescent Anti-IgG signal (using the un-infarcted contralateral ONs as baseline) revealed that PGJ_2_ treatment reduced post-infarct endogenous IgG levels in both the optic nerve head (lesion site), as well as the main body of the optic nerve (measured 2 mm behind the lesion site), compared with vehicle-treated infarcted nerves.

**Figure 5:**
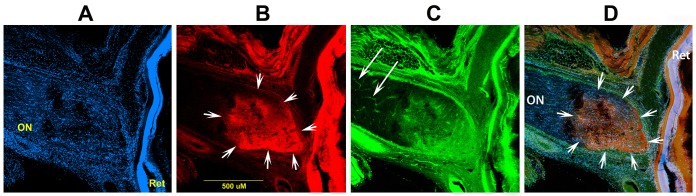
Confirmatory analysis of BBB breakdown in the anterior ON using low- and high-MW dyes. A. DAPI-counterstaining of the retina-ON junctional region. The ON and retina (Ret) are indicated. B. Rhodamine-3 kDa dextran label. Strong signal is present only in the anterior ON (the area of infarct; indicated by arrows), and the overlying retina. C. FITC-BSA label. The FITC signal is present in isolated capillaries in the posterior ON (long arrows), as well as a diffuse signal in the infarct region. D. Merged image, showing diffuse dextran-rhodamine and FITC-BSA signal overlap. Scale bar: 50 microns.

We confirmed the site of primary BBB breakdown via double dye labeling, utilizing both FITC-BSA (66 kDa) and a 3 kDa fixable dextran linked to rhodamine (Figure 5). Rhodamine-dextran (3 kDa) accumulates in the ON infarct region (Figure 5B; arrows), in a fashion similar to that seen with FITC-BSA (Figure 5C). The merged image reveals that both FITC-BSA and 3 kDa dextran accumulation is limited to the lesion in the anterior ON (Figure 5D, arrows), without leakage in the posterior ON, suggesting that gross BBB breakdown is limited to the region of the anterior ON without general loss of endothelial competency.

**Figure 6:**
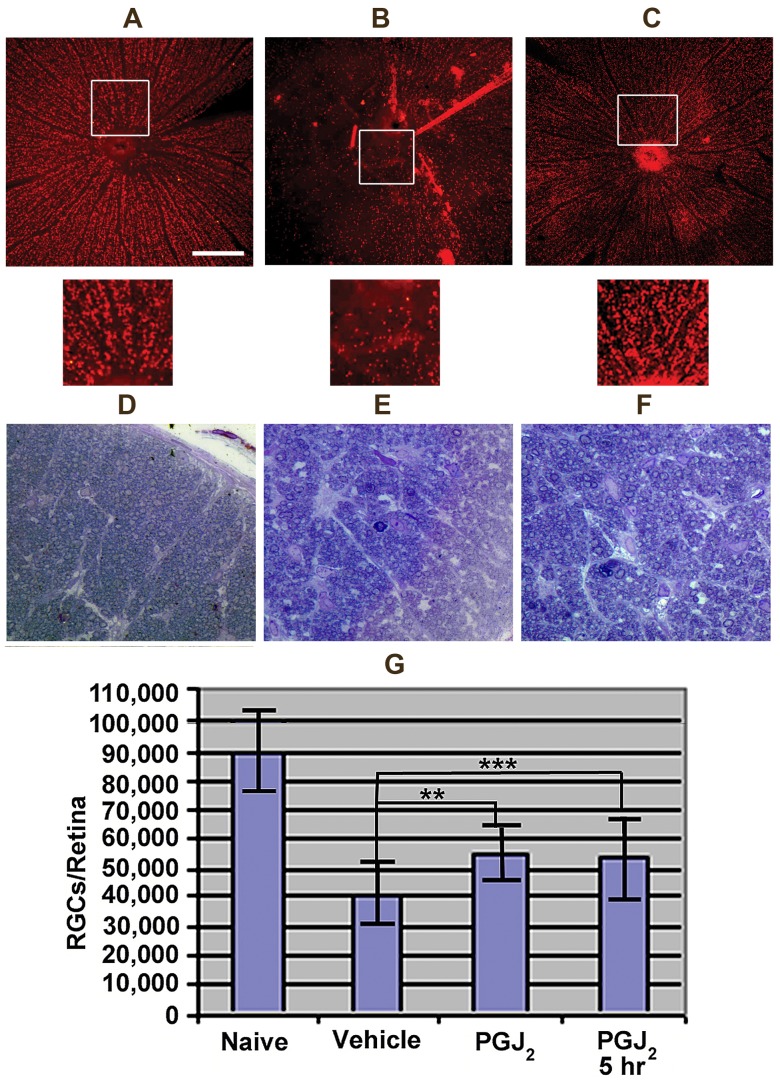
PGJ_2_-associated neuroprotection of ON and RGCs 30 days post-infarct. A–C: Brn3a stained retina flatmounts. Rectangles indicate high magnification retinal regions shown as insets immediately below. A. Control retina. Brn3a(+) RGCs are arranged evenly across the retina. B. Retina from an rAION-induced, vehicle treated animal. There is a regional loss of Brn3a(+) RGCs. C. Retina from an rAION-induced, PGJ2-treated animal. The retina shows smaller areas of RGC loss (compare C with B). D–F: Toluidine blue stained ON cross-sections. D. Naïve ON. Axons of varying diameters are packed into thinly septated (Sep) bundles (AxB). E. rAION-induced, vehicle treated animal. There is axonal loss, and increased septal thickness (Sep). F. rAION-induced, PGJ2-treated animal. Axonal loss is reduced, with thinner septae (Sep). G. Stereological quantification of RGCs in naïve, vehicle treated, and animals administered PGJ_2_ either immediately after or 5 hours post-infarct. Significance (ANOVA, p<0.05). Scale bars: A: 500 µm. D: 50 µm.

RGC neurons die after ischemic axonopathy [25]. Following rAION induction in the rat, >90% of RGC loss occurs by 30 days [26]. Unbiased stereology of Brn3a (+) RGCs was performed 30 days post-induction in naïve, vehicle, and PGJ_2_-treated animals (Figure 6).

Both immediate and delayed (5 hr post-induction) PGJ_2_ administration reduced rAION-induced RGC loss compared with vehicle controls (Figure 6A–C). The patterns of RGC- and axonal loss were also compared (Figure 6D–F) with those of naïve retina and ON.

RGCs in naïve retina are present as a monolayer of Brn3a (+) cells (Figure 6A, naïve, and inset, below). rAION-induced vehicle treated animals showed extensive regional loss of Brn3a(+) immunopositive cells (Figure 6B rAION-vehicle, and inset below). Systemic post-induction PGJ_2_ treatment improved overall RGC numbers at 30 days after infarct, compared with vehicle treated animals (Figure 6C, 15d-PGJ_2_, inset below).

We also compared the ON structure in the different treatment groups. Axons from naïve ONs are of different diameters, (Figure 6D, AxB) in thinly septated bundles (Figure 6D, Sep). ONs from rAION-induced vehicle-treated animals (Figure 6E) show diffuse as well as focal axonal loss and scarring (Figure 6E, AxB), with increased septal thickness (Figure 6E, Sep; compare with Figure 6D). In contrast, ONs of rAION-induced PGJ_2_-treated animals (Figure 6F) have structural characteristics intermediate to that of the naïve and vehicle-treated animals, with a milder diffuse axonal loss of axon bundles (Figure 6F, AxB), and fewer areas of complete loss (Figure 6F, compare with 6D and 6E).

Retinal stereological analysis 30 days post-infarct reveal extensive RGC loss in vehicle-treated animals (Figure 6G, vehicle: 55±12%, n = 12 animals), compared with naïve eyes (compare naïve and vehicle, Figure 6G). Systemic PGJ_2_ administration effectively reduced RGC loss when administered immediately post-induction (Figure 6G, PGJ_2_: 38%±14%, n = 10 animals). Since tissue edema is a gradual phenomenon, we hypothesized that PGJ_2_ could provide protection against the ischemia-related compartment effect even when administered at times later than the current maximum time limit used in thrombolytic interventions in stroke, i.e. 4.5 hours. To assess this we treated with PGJ_2_ 5 hours post-insult. PGJ_2_ is nearly as effective in reducing RGC loss at 5 hours post-induction as when administered immediately after rAION induction (Figure 6G, PGJ_2_ at 5 hours: 35±12% additional RGCs (n = 7 animals) vs. 38±14% additional RGCs in animals given PGJ_2_ immediately post-infarct). The neuroprotective effect was statistically significant at both treatment times, compared with vehicle (ANOVA, p<0.05).

### PGJ_2_ exerts ON neuroprotection by inhibiting the classical NFκB pathway

**Figure 7:**
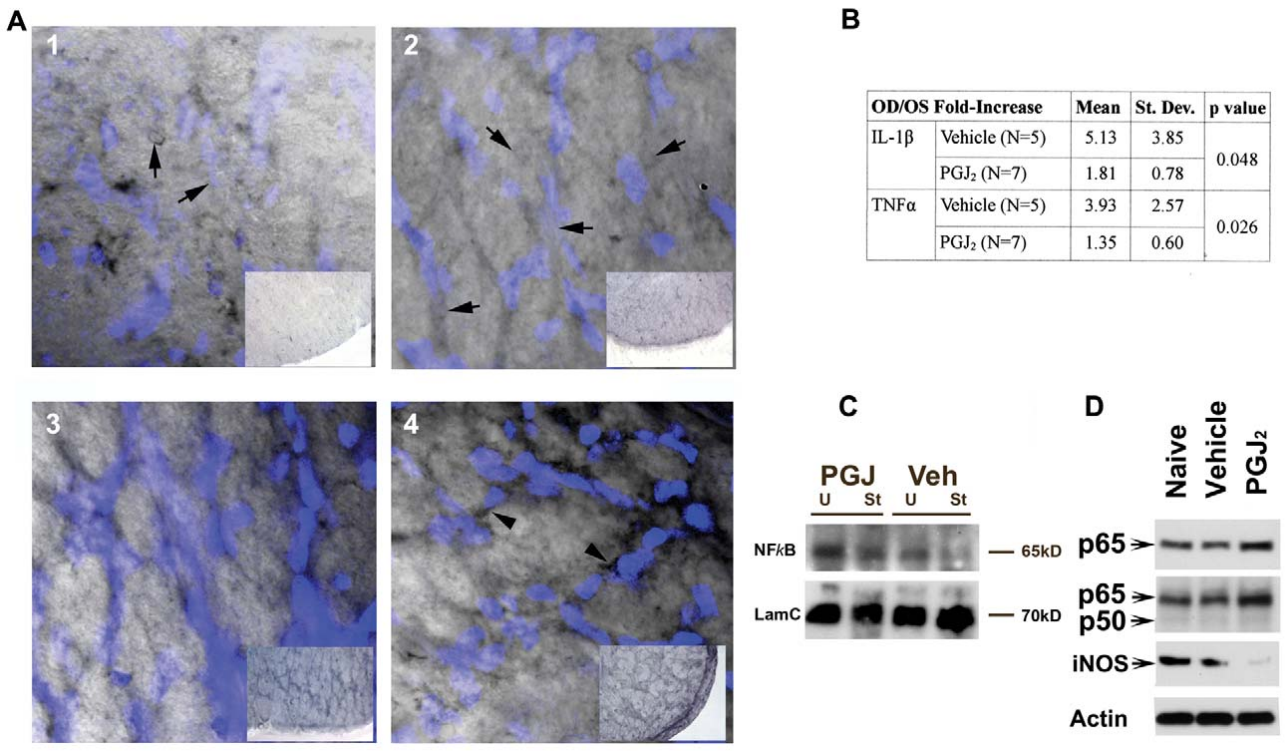
PGJ_2_ inhibits WM related NFκB activity. A. Ni-DAB quenched fluorescence analysis of NFκB expression and nuclear localization. ON sections developed and incubated identically to analyze relative Ni-DAB staining. Panels 1 and 2: ON cross-sections without PGJ_2_ treatment. Panels 3 and 4: ON cross-sections following PGJ_2_ treatment. Panel insets: low power micrographs showing relative ON NFκB staining. Panel 1: NFκB expression in un-induced, vehicle-treated ON. Note reduced DAPI intensity (inset). NFκB is present in nuclei, which have reduced visibility (arrows). Panel 2: NFκB expression in vehicle treated ON 1 day post-rAION induction. Increased intracellular NFκB expression (inset), relative to naïve ON, with reduced DAPI-stained nuclear visibility (arrows). Panel 3: NFκB expression in uninduced ON 1 d post-PGJ_2_ treatment. Note increased Ni-DAB signal (compare inset with panels 1 and 2), prominent DAPI-stained nuclei and perinuclear NFκB staining. Panel 4: NFκB expression in rAION-induced ON 1 day post-PGJ_2_ treatment. Note increased NFκB signal, compared with vehicle- or naïve tissue (compare inset panel 4 with panels 1 and 2), increased perinuclear NFκB accumulation (arrowheads). B. NFκB-associated gene expression in infarcted ONs with and without PGJ_2_. Comparison of infarcted- and contralateral nerves, expressed as R/L ratios, treated with vehicle or PGJ_2_, individual ON results (n = 12). C. ON western blot analysis: NFκB-subunit expression 1 d post vehicle- and PGJ_2_-treated animals. p65 signal increased in uninfarcted (U) PGJ_2_-treated ON and PGJ_2_-infarcted (I) ON, compared with infarct controls. Loading control: Lamin C (LamC). D. Western analysis: NFκB subunit- and NFκB-related (iNOS) expression in CNS (corpus callosum). p65 subunit expression with p65-specific antibody (Santa Cruz). Increased p65 signal seen in PGJ_2_- compared with vehicle-treated animals. Second row: expression using antibody with overlapping p65/p50 subunit specificity. Increased p65 is independent of p50 subunit levels. Third row: iNOS expression. PGJ_2_ treatment results in decreased white matter iNOS protein expression, compared with naïve or vehicle-treated animals. Loading control: β-Actin.

Previous studies of PGJ_2_'s mechanisms of action suggest that PGJ_2_ works by mixed mechanisms. These include peroxisome proliferator-activated receptor *gamma* (PPARγ) activation and NFκB inhibition. In order to identify ON-NFκB activation and its inhibition by PGJ_2_, we examined p65 (NFκB) expression and nuclear localization *in vivo* via a novel immunohistochemical technique (Figure 7A). Because κB sites (the target of the NFκB signaling complex) are numerous throughout the nuclear genome [27], NFκB cellular content is consequently quite high for a transcription factor. We localized the NFκB-p65 subunit by using immunohistochemical precipitation of nickel-diaminobenzidine (Ni-DAB), a black product that quenches 4′,6-diamidino-2-phenylindole (DAPI) fluorescence. Nuclei were stained *in situ* following NFκB immunolocalization with DAPI. The presence of p65-associated Ni-DAB quenches the DAPI signal relative to the concentration of intra-nuclear p65. Differences in nuclear p65 localization and concentration can therefore be evaluated by differential DAPI fluorescence when other immunostaining factors are kept constant (Figure 7A, panels 1–4).

We compared nuclear fluorescence levels between the different treatment strategies, as well as total cellular and nuclear Ni-DAB signal levels. Using this new technique, our data reveals that NFκB-p65 subunit expression is diffusely present in ON glial and vascular cells in the WM of the un-infarcted, vehicle-treated ON (Figure 7A, panel 1). There is considerable quenching of the DAPI nuclear signal even in naïve uninduced nerves (Figure 7A, panel 1 arrows). In vehicle treated animals 1 day post-rAION, there is a noticeable increase in overall p65 expression, with tissue darkening (Figure 7A, panel 2, compare insets, panels 1 and 2). There is also prominent p65 nuclear localization, with loss of nuclear DAPI staining (arrows, Figure 7A, panel 2). This is consistent with greater overall DAPI quenching (compare DAPI signal in Figure 7A, panels 1 and 2). In contrast, contralateral (uninduced) ONs of PGJ_2_-treated animals one day after treatment revealed increased p65 intercellular Ni-DAB signal, concurrent with strong DAPI fluorescence (Figure 7A, panel 3), compared with naïve ON (compare vehicle to naïve in panels 3 and 1, and compare insets). rAION-induced ONs in PGJ_2_ treated animals also show increased intracellular p65 signal (Figure 7A, panel 4 inset), but conversely, also show increased DAPI nuclear fluorescence (Figure 7A, panel 4; note blue color intensity). These data suggest that while total cellular p65 increases in infarcted PGJ_2_-treated ON tissues, there is decreased p65 nuclear localization. This result is supported by the strong perinuclear p65 signal in the same tissues (arrowheads, Figure 7A, panel 4), indicating that NFκB is sequestered in the cytoplasm in these cells. Thus, PGJ_2_ administration results in strong p65 up-regulation independent of rAION treatment. ON infarct also results in additional p65 up-regulation. The increased p65 expression is not translocated to the nucleus in PGJ_2_-treated animals.

To independently confirm this finding, we compared well-documented p65 downstream transcript targets via qRT-PCR (Figure 7B). ON total RNA was isolated from rAION-induced and contralateral un-induced nerves of individual animals 1 day post-rAION. Animals were treated with either vehicle, or PGJ_2_ (n = 5/group). First strand cDNA was prepared by random priming and analyzed for IL-1β and TNFα expression. IL-1β and TNFα mRNA upregulation occurred 1 day post-infarct induction (Figure 7B). PGJ_2_ administration significantly reduced ON levels of both cytokines (Figure 7B; Students t-test, p<0.05). These results suggest that PGJ_2_ administration inhibits the classical NFκB-associated inflammatory pathway activated after ischemia.

We also evaluated NFκB-related p65 protein levels using western blot analysis (Figure 7C). Protein homogenates were prepared from the first 2 mm of ON and ONs were pooled (n = 3) to achieve sufficient detection concentration. Four conditions were evaluated: 1 day post-rAION induction (I) or uninduced (U) ONs treated with either PGJ_2_ or vehicle (veh). p65 expression increased slightly in vehicle treated ON post-infarct, compared with uninduced (naïve) control ON. PGJ_2_ administration greatly increased p65-ON expression, regardless of rAION status, compared with naïve or vehicle (Figure 7C; compare PGJ lanes with vehicle and naive).

We confirmed PGJ_2_'s effects on p65 expression in corpus callosum (CC); another WM tract, by western analysis (Figure 7D). Tissues were dissected from both naïve- and 1 day post-PGJ_2_-treated animals. Western analysis revealed PGJ_2_-associated upregulation of p65 (Figure 7D, p65, compare naïve and vehicle-treated with PGJ_2_-treatment). Analysis using a polyclonal antibody that recognizes both p50 and p65 subunits also revealed p65 upregulation only in PGJ_2_-treated CC (Figure 7D; p50/p65 row). PGJ_2_ treatment also reduced inducible nitric oxide synthase (iNOS), a protein known to be controlled by NFκB activity (Figure 7D, iNOS), compared with naïve or vehicle treated animals. All these data confirm that PGJ_2_ administration upregulates p65 and blocks it from entering the nucleus in both CNS white matter structures, the ON and CC.

### PGJ_2_ is not toxic to oligodendrocytes *in vivo*

**Figure 8:**
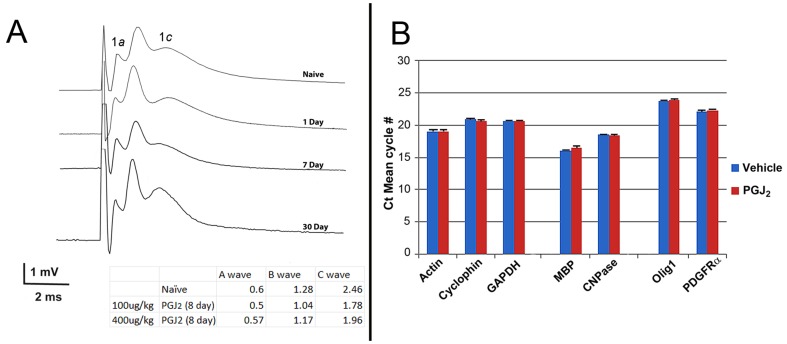
Analysis of PGJ_2_-related oligodendrocyte toxicity. A. Compound action potentials (CAPs) from naïve ON and ON 1, 7, and 30 days after a single treatment of PGJ_2_ (100 µg/kg).No significant changes from naïve ONs were seen. Inset: Wave amplitude maxima of individual axon components, from the ONs of animals given either 100 ug- or 400 ug/kg PGJ_2_ prior to testing TEM analysis of naïve and 30 days post 400 µg/kg PGJ_2_. No differences are detectable in axonal appearance, myelination, or overall structure. Low power bar = 500 nm High power bar = 100 nm. C. Evaluation of oligodendrocyte (NG2(+)) precursors 30 days post-PGJ_2_ administration (100 µg/kg). No differences in NG2(+) cells were seen in the ON.

**Figure 9:**
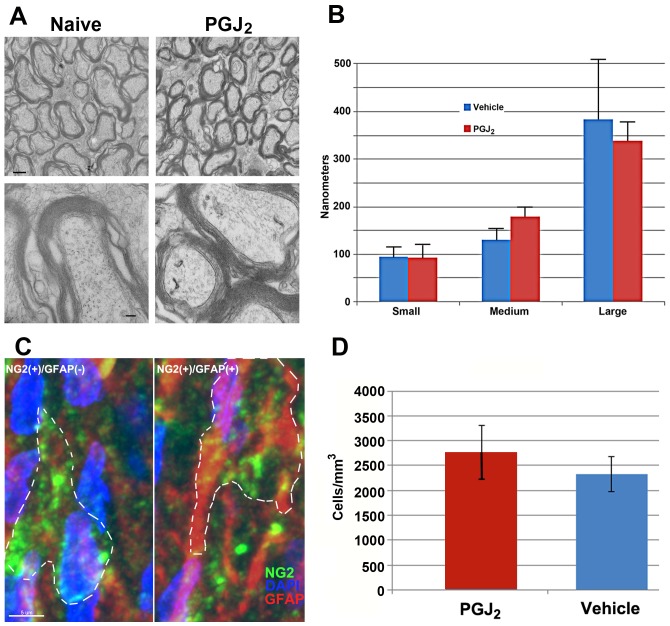
Oligodendrocyte- ultrastructural and -precursor analysis in vehicle- and PGJ_2_-treated ONs. Animals were euthanized 30 days post-treatment and tissues prepared for either TEM (A and B) or immunohistochemistry (C and D) (see methods). A (top panels): low magnification of ON from either vehicle or PGJ_2_-treated animals. (bottom panels): High magnification of ON from vehicle- and PGJ_2_- (blue bars) treated animals. B. Myelin thickness graph from the ONs of vehicle- (red bars) and PGJ_2_- (blue bars) treated animals (Figure 9B). Axons from three distinct size group (small, medium, and large axons/group) were evaluated (n = 5 axons/group). Mean thickness is similar in each group for both conditions. Mean ±sd. C. Confocal analysis of NG2(+)/GFAP(−) (presumed oligodendrocyte precursors) and NG2(+)/GFAP(−) (Presumed astrocyte precursors) from the ON of a naïve animal. Both NG2(+) (Cy5 secondary label, seen in green)- GFAP(−) and GFAP(+) (Cy3 secondary label, seen in red) cells are scattered throughout the ON in naïve- and PGJ_2_- treatment groups. D. Stereological analysis of NG2 expressing cells in vehicle- and 30 day post-PGJ_2_ treated ONs (n = 5 nerves/group) showed similar numbers of NG2(+)/GFAP(−) cells per unit volume. p>0.05, Mann-Whitney U test.

We evaluated PGJ_2_'s long term-associated *in vivo* effects on neuronal and oligodendrocyte function by four methods: 1) Analysis of myelinated axonal conduction speed via compound action potential (CAP) analysis (Jimura 33–37). This was compared to un-infarcted naive- and PGJ_2_-treated ONs (i.v. 100 µg/kg PGJ_2_ at 1, 7 and 30 days post-administration, (Figure 8A). 2) qPCR analysis of mRNA from animals given 100 ug/kg PGJ_2_ eight days prior to analysis (n = 3 animals/group; total 6 ONs/group) (Figure 8B). 3) TEM-ultrastructural analysis and quantification of myelin thickness of ON-axons from naïve *vs.* PGJ_2_-treated (100 ug/kg) animals (Figure 9A and B). 4) Quantitative analysis of the NG2 (+) expressing oligodendrocyte precursors (NG2(+)/GFAP(−)) in ONs from naïve vs. PGJ_2_-treated (100 ug/kg) animals 30 days post-treatment (Figure 9C and D).

ON-CAPs from naïve animals generate three amplitude peaks corresponding to the relative axon diameters present in rat ON (Figure 8A-naïve). Slight apparent differences in ON transmission speed were seen between naïve and treated animals 8 d post-treatment) animals but interneural differences are also present when different naïve animals are compared with each other (D. Weinreich, unpublished data) (see inset, Figure 8A). Slight CAP variations were also apparent in ONs from animals given 400 ug/kg PGJ_2_ eight days prior to testing, compared with naïve controls (compare naïve, 100 and 400 ug/kg values: inset, Figure 8A). Thus, data from PGJ_2_-treated ONs were all within the naïve range group (D. Weinreich, unpublished data).

q-PCR of ON mRNA from animals treated 8 days post-100 ug/kg also revealed little change in housekeeping genes, or myelin-related genes typically associated with mature oligodendrocytes (MBP and CNPase; Figure 8B). Analysis of genes associated with immature- or precursor oligodendrocyte function (Olig-1 and PDGFRα; Figure 8B) also showed little change in treated animals, compared with vehicle controls.

TEM-analysis of ON ultrastructure was compared from naïve- *vs* PGJ_2_-(100 ug/kg) treated ONs 30 days after administration (Figure 9A). Axons in the naïve ON are tightly packed into septated bundles that are most easily seen at low magnification (Figure 9A top panels). Axonal neurofilaments and laminated oligodendrocyte myelin are clearly visible at higher magnification (Figure 9A, lower panels). No ultrastructural changes were qualitatively detectable in the ON-myelin sheaths of animals treated with PGJ_2_, compared with naive. We also quantified myelin sheath thickness in the ON axons from animals 30 days post-PGJ_2_ administration (Figure 9B, as measured by total thickness of the myelin sheath at the most compact point detectable). There was little difference in average myelin thickness of any axon type (small, medium, or large diameter fibers) (Figure 9B; compare mean myelin thickness for each group) in the rat ON.

Oligodendrocyte precursors have been reported to be more sensitive to PGJ_2_ toxicity *in vitro* than mature oligodendrocytes [20]. We evaluated NG2(+)/GFAP(−) oligodendrocyte precursor cells 30 days post-treatment, in the ONs of naïve- and PGJ_2_-treated (100 µg/kg) animals (Figure 9C) [28]. In the rodent ON, NG2(+) cells that are either GFAP(+) or GFAP(−) are distributed throughout the nerve sections (Figure 9C). Similar NG2(+) expression patterns were seen in ONs from naïve and PGJ_2_-treated animals (30 days post-treatment). Stereological analysis of ON-NG2(+) cell numbers revealed similar numbers of NG2(+) cells in both vehicle- and PGJ_2_-treated animals 30 days post-administration (Figure 9D). Thus, PGJ_2_ does not result in the long-term loss of oligodendrocyte precursors *in vivo*.

## Discussion

Our study shows that PGJ_2_ reduces ON infarct damage when administered immediately after rAION injury and even 5 hours post-injury. PGJ_2_ therefore provides an extended window of stroke treatment opportunity, as measured by 30 day neuronal survival. An important mechanism of action appears to be edema reduction, which can improve infarcted ON tissue perfusion after injury.

The current ON-infarct model demonstrates many of the responses of CNS white matter-specific damage, including pro-inflammatory signals such as TNFα [29] and IL-1β [30]. These cytokines are associated with increased BBB permeability [31]. Tissue edema in the ON-restricted space can produce a compartment syndrome that compromises otherwise uninvolved capillary lumens in the infarct penumbra. Compartment syndromes have been demonstrated to play a major role in the extension of damage in lesions involving grey matter [32], [33].

In the ON-infarct model, direct blockade by platelet-fibrin thrombi provide a relatively small contribution to overall ischemia. Large ON vessels occasionally showed small, adherent thrombi that did not block the lumen. Most of the perfusion loss appears to result from progressive edema, which further restricts capillary perfusion in the local vascular bed. This is demonstrable by the minimal capillary dropout at 4 hours, either with or without treatment, compared with the much greater loss of perfusion at one day post-induction (see Figure 2). The progressive edema in vehicle-treated animals compromises additional capillaries in otherwise unaffected tissue. These progressive decompensatory mechanisms continue after 4 hours, evidenced by both adequate perfusion of the ON at 4 hours post-rAION, and PGJ_2_'s ability to block edema when given five hours post-induction. The current model provides an advantage in that development and reduction of WM edema can be directly observed.

In addition to its antagonistic effects on NFκB, PGJ_2_ is a perioxisomal proliferator-activated receptor *gamma* (PPARγ)- and Nrf2-agonist [34], [35]. All of these distinct activities in white matter may contribute to PGJ_2_'s anti-inflammatory properties. The calculated initial blood concentration after administration of 100 µg/kg PGJ_2_ injection is approximately 4.9 µM, based on rat blood volume [36]. Similar prostaglandins can enter all rat tissues [37], but with significantly reduced CNS penetration relative to skin and internal organs. Based on this, we presume that the exposure of endothelial cells is likely to be much higher than that seen by other CNS components. Thus, it is not surprising that the CNS vasculature is a primary site of PGJ_2_ interaction.

While PGJ_2_ administration reduces expression of the NFκB classical pathway-related genes TNFα and IL-1β, there are a large number of NFκB-related transcriptional sites present in the genome. This large copy number and the strong intracellular NFκB expression enables us to utilize immunohistochemical NFκB-Ni-DAB quenching of DAPI nuclear signal. Our data show increased p65 cellular signal following infarct, in vehicle treated animals, with typical nuclear localization, which is coupled to the increase in downstream transcription of related genes. This was confirmed by western analysis. However PGJ_2_ administration by itself elicits even more intracellular p65 expression than ON infarct alone. This is demonstrable by increased Ni-DAB signal, in both injured and uninjured ON, of PGJ_2_-treated animals. But while there is increased NFκB staining in PGJ_2_-treated animals, there is reduced DAPI quenching, detectable by both increased DAPI nuclear fluorescence in PGJ_2_-treated ONs and increased NFκB perinuclear signal. These data confirm that PGJ_2_ treatment blocks p65 translocation to the nucleus. NFκB activation is typically associated with IL-1β and TNFα upregulation in CNS infarct, but is significantly reduced with PGJ_2_ treatment, despite an overall increase in cellular NFκB. Thus, the net effect of PGJ_2_ in vivo is to reduce p65-mediated signaling by retaining it in the cytoplasm.

To confirm the generality of PGJ_2_'s mechanisms of action in CNS white matter, we compared protein expression in CC of naïve and PGJ_2_-treated animals (Figure 7D). CC responses to PGJ_2_ are similar to those seen in ON. Interestingly, CC-p65 subunit levels increase while the p50 subunit of NFκB remains constant. This behavior has previously been observed in rat CNS endothelial cells after *in vivo* hypoxia/reperfusion injury [38]. Upregulation of iNOS expression following ON infarct is a relatively late phenomenon [39], which may be related to the post-infarct macrophage influx seen in rAION [24], and confirmed for clinical NAION [40].

Significant controversy revolves around PGJ_2_'s potential neurotoxicity. A number of reports have focused on *in vitro* administration to cell cultures [20], [41]. These systems can respond differently in their sensitivity compared to what is seen *in vivo*. PGJ_2_ also may be neuroprotective at low concentration and induce apoptosis at higher doses [17], [20]. Concentration at the site of action may also be important, since prostaglandins can be highly lipophilic and be absorbed by capillary membranes. We did not find in *in vivo* PGJ_2_ oligodendrocyte toxicity, as measured by ON-myelin gene expression, alterations in myelin thickness, toxicity to oligodendrocyte precursors (both by qRT-PCR and immunohistochemistry), or changes in overall ON function when PGJ_2_ was administered in doses similar to those used in our neuroprotection assays. These data suggest that systemically administered PGJ_2_, when given in doses that produce measurable neuroprotection against focal axonal ischemia, is relatively nontoxic to ON white matter *in vivo*.

A major problem facing clinicians treating CNS infarcts is the current short treatment time window; currently limited to 4.5 hours or less for ‘clot buster’ drugs [42]. This may be due to the greater sensitivity of the gray matter-neuronal cell body component to ischemia. Since the majority of ON capillaries in the ON-white matter CNS tract are still patent at 4 hours post-induction, the time window for WM-infarct treatment may be much longer than currently considered. WM infarct therapy may also be complementary to other neuroprotective strategies. PGJ_2_'s neuroprotective effect is seen even when given 5 hours post-induction. The differential nature of CNS tissue injury following infarct, coupled to the large WM contribution to human brain mass, suggest that a neuroprotectant such as PGJ_2_ that protects at a vascular level should be considered as a useful addition to grey matter neuroprotective approaches in order to achieve a maximally effective therapeutic rescue for infarcted CNS tissue.

### Disclosure

The use of PGJ_2_ for NAION has been granted US patent #8,106,096 to the University of Maryland-Baltimore.
